# The Walla Emotion Model (WEM): A New Terminology Redefining Affective Dysregulation in Clinical Psychopathology

**DOI:** 10.3390/brainsci16050512

**Published:** 2026-05-11

**Authors:** Peter Walla

**Affiliations:** 1 Freud CanBeLab, Faculty of Psychology, Sigmund Freud Private University, Freudplatz 1, 1020 Vienna, Austria; peter.walla@sfu.ac.at; 2 Faculty of Medicine, Sigmund Freud Private University, Freudplatz 3, 1020 Vienna, Austria; 3 School of Psychology, Newcastle University, University Drive, Callaghan, NSW 2308, Australia

**Keywords:** affection, non-conscious processing, startle reflex modulation, cognition, emotion, feeling, affective processing, cognitive processing, borderline personality disorder, major depressive disorder, clinical diagnosis, clinical treatment

## Abstract

**Featured Application:**

The newly introduced terminology is considered helpful in terms of diagnosing clinical conditions as well as explaining their various aspects that have been summarized as “emotion dysregulation”. It is suggested to replace “emotion dysregulation” with “affection dysregulation” and to use the term feeling and emotion separately (as suggested by the WEM).

**Abstract:**

The scientific pursuit of understanding human “emotion” has historically been plagued by a fundamental lack of conceptual consensus. Researchers, clinicians, and the lay public frequently utilize terms such as “emotion,” “feeling,” “affect,” and “mood” as interchangeable synonyms, creating a linguistic ambiguity that hampers both experimental precision and diagnostic validity. In response to this “umbrella term” crisis, the Walla Emotion Model (WEM), also referred to as the ESCAPE Model (Emotions Convey Affective Processing Effects), introduces a redefined and distinct terminology designed to disentangle the neurophysiological, experiential, and behavioral components of affective phenomena. The essence of this new model is the removal of the umbrella aspect from the term emotion and defining “emotion” strictly as behavioral output, and “feeling” as the conscious perception of released neurochemicals, both resulting from non-conscious affective processing. By doing so, the WEM provides a logical, clear, and easy-to-apply terminological lens for diagnosing, communicating, and treating clinical conditions that include what has previously been termed “emotion” dysregulation. When “emotion” is used as an umbrella term, it depends on the school one follows how one would explain such clinical conditions. The most critical argument for introducing the WEM is that each prior school has had its focus on another set of phenomena that generate an “emotion”. The WEM terminology provides a clear separation of brain activity, subjective experience, and expression regarding affective phenomena. Various clinical conditions that include “emotion” dysregulation exist; however, to highlight the potential benefits of the WEM, the current essay has its focus on two of the most frequent conditions, namely Borderline Personality Disorder (BPD) and Major Depressive Disorder (MDD). The goal is to provide an analysis of the WEM architecture, evaluating its utility in clinical neuropsychology, and delineating its theoretical advantages when combined with traditional categorical and dimensional models. However, it is important to emphasize that this essay is only theoretical. It does not include any direct empirical support, but it suggests the replacing of existing terminology with WEM terminology.

## 1. Introduction

Probably the best argument for introducing a more precise terminology to separate the different aspects of affective phenomena is because the various existing “emotion” definitions vary in what they all include in the meaning of the term emotion (umbrella aspect). Depending on the theory (or school), these could be physiological, experiential, expressive, and even cognitive aspects. Thus, using “Emotion” as an umbrella term does not describe “emotion” dysregulation very precisely. In fact, depending on the emotion theory, “emotion” dysregulation has varying meanings and leaves both the patient as well as the treating professionals without a clear understanding about the actual problem. At this stage, it is important to mention that this essay is only conceptual; however, it is believed that the introduced emotion model framework indeed provides a useful terminology for the benefit of both patients and clinical psychologists (as well as therapists and psychiatrists). The proposed terminology is meant as an operational simplification as well as a broader theoretical redefinition. For a comprehensive and neurobiological grounded explanation, see the original article [1]. However, the following section provides a short overview of the Walla Emotion Model (WEM) [1], allowing for a good impression to evaluate its potential benefits.

### The Foundations of the Walla Emotion Model (WEM)

The WEM [1] is based on the neurobiological notion that the brain’s primary function, beyond maintaining homeostasis, is to produce adapted behavior to ensure optimal navigation through the environment, best possible social interactions, and survival in general [2]. To achieve this, the brain processes information through two distinct yet interacting channels, a cognitive and an affective one. While cognitive processing is concerned with semantic features like identifying “what” an object is, affective processing, on the other hand, is evaluative, determining “how” an object relates to the individual’s survival and well-being [2,3]. The WEM organizes the evaluative “how is something” aspects of information processing into three separate phenomena. First, affective processing (i.e., subcortical evaluative neural activity), defined as the rapid, primarily non-conscious neural activity, evaluates internal and external stimuli based on valence (pleasant or unpleasant) and arousal (the intensity of the stimulus). This processing occurs in evolutionarily ancient subcortical structures (mainly the limbic system), which are shared across a wide range of vertebrate species [4,5]. Because these structures evolved long before the cortical mechanisms associated with language and higher-order cognitive processing, affective processing (also referred to as affection) operates independently from conscious awareness and cognitive oversight. Even though cognition, once evolved, can influence affection, it is considered helpful to primarily understand these two processing systems as independent and separate.

The basic level of “raw” affective processing serves as the primary driver of behavior. Interestingly, there is reason to believe that this basic and evaluative information processing network might have its origins in the sense of olfaction in terms of evolution [6]. While this is not overly important within the frame of this essay, it is still considered potentially helpful for future investigations on psychological disorders that include some form of affective dysregulation (in response to the here-introduced WEM, the term “emotion” dysregulation is replaced with the term “affection” dysregulation). Anyway, affective processing initiates predispositions for action (such as approach or avoidance) before the individual is consciously aware of the stimulus triggering the response (some emotion models would state that an emotion is the driver for motivated behavior, but in those models the term emotion is associated with another meaning). In early human childhood and in non-human animals, behavior is guided almost exclusively by this affective mechanism [2]. Cognitive capacities need more time to develop during ontology (i.e., lifespan), while affection develops earlier in life. This explains the more affection-based behavior in early human childhood. According to the principle, what comes later in terms of human development goes first [7], it also means that due to cognition being more vulnerable and thus malfunctioning prior to affection-problems, elder people’s behavior gets again more affect-based (similar to early childhood). While this is also not overly important for the current essay, the clinical significance of the level of affective processing is profound. It suggests that many dysregulated behaviors seen in psychiatric populations are the result of subcortical evaluations that occur entirely outside the patient’s conscious control or understanding [8].

Feelings are defined by the WEM as conscious perceptions that arise when affective processing reaches a specific “threshold” level, as in neural activity reaching certain graded potential values [9,10], resulting in phenomena often referred to as bodily responses. When subcortical evaluative activity becomes sufficiently intense, it triggers the release of neurochemicals, including various neurotransmitters and hormones, into the bloodstream and across synaptic junctions (i.e., the bodily responses). The respective changes in the body’s internal milieu are perceived consciously as a “feeling” [2]. In this framework, states like “fear,” “anger,” “happiness,” or “sadness” are classified as feelings, not emotions. A feeling is the “what it’s like” of an internal state—the felt experience, or in other words, the perceived physiological consequences of supra-threshold affection. Critically, because feelings require consciousness and are filtered through the individual’s subjective perception, they can be influenced by cognitive schemas or cultural expectations, leading to what has been described as “cognitive pollution” [11]. This explains why patients in clinical settings may mislabel their internal states or report feeling nothing (emptiness) even when physiological data indicates high affective activity.

The most distinctive and technically disruptive aspect of the WEM is its redefinition of the term “emotion” [1], which the WEM describes as an observable behavioral output resulting from muscle contractions that communicate a felt state to others. Such outputs include facial expressions, vocalizations (such as a gasp or a sob), body posture, and gestures, among many more behaviors that convey feelings. Crucially, according to the WEM, an emotion is a signal to the external world, not an internal state or an information-processing mechanism. For instance, the feeling of “fear” is internal and subjective, whereas the “scared face” (widened eyes, open mouth) is the “emotion”. Crucially, the underlying neural activity for both phenomena is affective processing. Interestingly, this framework allows for the existence of “voluntary emotions”, which are emotion behaviors produced to feign a feeling for various reasons, such as in social settings (masking). Those are neurologically distinct from “involuntary emotions” triggered directly and automatically by affective processing. This separation is vital for clinical diagnoses, as it permits the identification of desynchrony between what a patient feels and what they express [12]. In fact, in some clinical cases, even involuntary emotion behavior might not be a 1:1 reflection of its underlying affective processing and/or feeling level.

**Figure 1:**
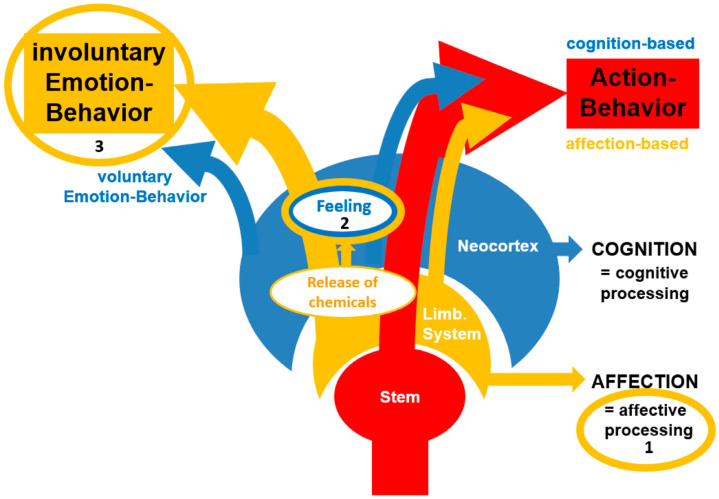
The Walla Emotion Model (WEM) (or the ESCAPE Model (Emotions Convey Affective Processing Effects)). First, (1) affective processing provides evaluative information to support decision-making for the production of action behavior (behavior guidance). Second, (2) supra-threshold affective processing by the limbic system leads to released chemicals controlled by hypothalamic involvement (neurotransmitters and hormones). From this, feelings arise by an organism that is capable of conscious experience. (3) In response to affective processing, involuntary emotion behavior is also produced to communicate a feeling to others. Interestingly, at least humans are capable of displaying voluntary emotion behavior, which becomes evident in social masking (taken from Walla et al. [1]).

See Table 1 for a list that summarizes the three components of the WEM and Figure 1 for a schematic visualization. For a more detailed neurobiological explanation, it is again recommended to see the original article [1] that provides a much more detailed explanation of these three affective phenomena, including their suggested evolutionary origins.

## 2. Clinical Benefits

While the list of psychological disorders that are associated with some sort of dysregulated affection is long, this section pays attention to two of the most dominant conditions, including affection dysregulation. As mentioned above, before going into any details, it is considered important to emphasize again that, in response to the herein introduced new terminology, the term “emotion dysregulation” is no longer used due to its confusing and misleading nature. This essay suggests using the term “affection dysregulation” as a more precise label. Anyway, the two conditions that are critically reflected on here in the context of the WEM are Borderline Personality Disorder (BPD) and Major Depression Disorder (MDD).

### 2.1. Borderline Personality Disorder (BPD)

Borderline Personality Disorder is a condition defined by profound instability in affection, behavior, identity, and interpersonal relationships [13,14]. BPD affects 2–5% of the population and it is one of the most frequent personality disorders [15]. According to Duberstein and Conwell (1997) [16], diagnosed BPD is associated with severe functional impairments and quite high suicidal death rates (5 to 7%). This mental disorder poses a significant high health concern regarding physician involvement and hospitalization [17].

Most importantly, in the current context of this essay, traditional models, such as Marsha Linehan’s biosocial theory [18], emphasize “emotion dysregulation” as the core of BPD, resulting from a combination of biological vulnerability and an invalidating environment. As already mentioned at the beginning of the introduction, the best argument for the current proposal is the typical umbrella nature of the term emotion. If one would try to explain BPD-related “emotion dysregulation” by using the variety of, to-date, existing emotion theories, it would very soon turn out to be rather difficult, if not even confusing and potentially dangerous, because of inevitable misunderstandings and discrepancies. Understanding the term emotion as an umbrella term is the problem. As a result of this, one cannot know which aspect (or aspects) of “emotion” is (are) dysregulated, which in turn hinders communication regarding a diagnosis as well as treatment strategies. This obvious difficulty is taken as the most important argument for the WEM to be introduced into clinical psychopathology. The WEM enhances the biosocial theory by providing a more granular map of where the dysregulation occurs while also providing a set of clear labels to name the different affective phenomena that are affected. By using the terminology of its framework, the “affection dysregulation” characteristics of BPD are primarily defined as hyper-reactive subcortical affective processing. For individuals with BPD, the subcortical “alarm system” (mainly the amygdala) evaluates stimuli with extreme negative valence and high arousal, even when those stimuli are objectively neutral or mildly challenging. This hyper-reactivity occurs at a non-conscious level, meaning that the patient is “triggered” before their conscious has awareness of what has happened. The non-conscious nature of affective processing leads to a biological trap. Because it is subcortical and has no direct link to the language centers of the cortex [2], the patient cannot explain why they are suddenly in distress. They simply experience the result of a massive chemical cascade that reaches the “feeling” threshold almost instantaneously. This provides a neurobiological explanation for the “exposed nerve” sensation reported by many BPD patients [18].

Research on the Emotional Cascade Model [19,20] suggests that BPD symptoms are driven by a positive feedback loop of rumination and negative affect (affect would be replaced with affection according to the WEM). Through the lens of the WEM, this can be seen as sustained supra-threshold affective processing. Rumination (a cognitive/cortical process) re-triggers the subcortical affective system, keeping the body in a constant state of chemical release. In healthy humans, “affective processing” and “cognitive processing” are integrated to help us navigate through the world and adapt our behaviors in the most optimal way. However, in what is usually called an “emotional” cascade (this term also needs adjustment according to the WEM), those two functions become a maladaptive whole. Cognitive rumination and negative affection become a tight unit, further increasing levels of distress.

When these conscious feelings become unbearable in response to hyper-active affective processing, the individual produces involuntary “emotions”—behavioral outputs—to communicate the internal state. In BPD, these emotions are often dysregulated too, such as self-harming behaviors or explosive outbursts. The WEM posits that these behaviors are communicative signals of distress (emotions), but also serve as potent physical stimuli to “distract” the brain from the internal feelings generated by the rumination cycle. By identifying self-harm as a communicative “emotion” rather than the disorder itself, clinicians can shift focus toward regulating the underlying “affective processing” and the “feelings” that the behavior is attempting to express. While this is all theoretical speculation, it could provide new insights into how to treat this particular disorder. With respect to therapeutic implications, the WEM’s distinction between feelings and emotions supports the structure of Dialectical Behavior Therapy (DBT) [21]. DBT skills such as mindfulness and “Observe and Describe” are essentially exercises in training the conscious cortical self (Me2) [22,23,24,25,26] to accurately perceive the subcortical affective signals that could, in terms of self-referential processing, be related to the so-called Me1 [24] without being overwhelmed by them. Furthermore, understanding that emotions (according to the WEM they are behaviors) can be voluntary or involuntary [1] helps therapists distinguish between a patient’s genuine distress and social masking (or camouflaging), which is a common challenge in BPD treatment [27]. In summary, the terminology suggested by the WEM is considered more precise than by other emotion theories; mainly, because it reduces the meaning of the term “emotion” to “communicative behavior” instead of using it as an umbrella term including various sub-phenomena, not all of which are necessarily dysregulated in the first place (but in consequence).

### 2.2. Major Depressive Disorder (MDD)

Major Depressive Disorder (MDD) involves persistent low mood and a loss of interest or pleasure, often accompanied by cognitive and physical symptoms [28,29]. Also, here, the aim is to apply the WEM in order to potentially get a more detailed opportunity to label the dysregulated phenomena of this clinical condition and thus perhaps offer more accurate treatment options. The WEM offers specific advantages in understanding the desynchrony between internal states and external behaviors in MDD. Clinical neuroscience research utilizing Event-Related Potentials (ERPs) has demonstrated that individuals with elevated depressive symptoms fail to effectively suppress task-irrelevant negative stimuli [30,31]. Thus, suppression failure and an implicit negativity bias could be reported with objective neurophysiological measures. In WEM terms, subcortical affective processing in depressed individuals is characterized by a persistent “negative-tending” evaluation of the environment. This has also been shown with Startle Reflex Modulation (SRM) [32,33]. When viewing positive images, depressed patients can still self-report positive impressions; however, their raw affective processing (non-conscious) shows significantly more negative processing compared to healthy controls. The implicit bias thus occurs regardless of what the patient reports back on a cognitive level. A depressed patient might also describe their state as “numb” or “empty”—a lack of conscious feelings—while their brain continues to exhibit heightened affective negativity at the subcortical level. The WEM emphasizes that “affective processing can happen without generating an emotion” (observable behavior) [1]. This “flat affect” (also this term should be reconsidered) is a classic symptom of MDD, but the WEM clarifies that it is a lack of communication (emotion), not necessarily a lack of processing.

Depression is often associated with a deficit in positive affection. The WEM suggests that in chronic depression, the threshold for triggering positive “feelings” (via chemical release) may be elevated, or the affective processing of pleasant stimuli may be too weak to reach the supra-threshold level. This provides a neurobiological basis for anhedonia [34]. The brain “knows” a stimulus is objectively pleasant (cognition), but the affective processing fails to trigger the chemical cascade necessary to create the conscious “feeling” of pleasure. Because language (cortical) has no direct access to subcortical affective processing, self-reports of depression can be inaccurate or influenced by social desirability or the above-mentioned cognitive pollution. The WEM advocates for the use of objective technologies to measure the progress of depression treatment. For example, objective measures like Startle Reflex Modulation (SRM) can reveal improvements in the underlying affective circuitry before the patient consciously “feels” better. SRM is considered superior to typical brain imaging tools, especially in the current context. For instance, applying this totally non-invasive method not only showed that raw affective processing in depressed people has a significant negativity bias towards positive stimuli, but also that antidepressant treatment with selective-serotonin-reuptake-inhibitors (SSRIs) can change this back to normal after weeks of treatment. The respective delay of a measurable effect in turn goes along with the reported delay of felt improvements [32]. Because of its highly appreciated value, SRM is explained further below.

### 2.3. Potential Operationalization of the WEM

In order to get a better impression of how affective processing, feelings, and emotions can be identified and measured in practice, the following paragraph is meant to provide an imaginary example. In a study applying the WEM to clinical populations, one would be able to distinguish between non-conscious evaluative reactions (i.e., affective processing including the release of chemicals), conscious experiences (i.e., feelings), and behavioral output (i.e., emotions) to map the distinct profiles of Borderline Personality Disorder (BPD) and Major Depressive Disorder (MDD). Affective processing could be quantified by Startle Reflex Modulation (SRM), while feelings can be collected via self-report. Emotions, on the other hand, could be filmed and analyzed via facial expression software, or they could be measured more precisely via electromyography (EMG). This would allow the quantification of muscle contraction changes in response to changes in facial expressions very accurately. In addition, any behavior coding would add to emotion-behavior data.

In the case of BPD, by integrating these measures, a respective study would be able to test the hypothesis that BPD patients exhibit heightened non-conscious affective processing to relatively low-level affective triggers (e.g., slightly angry face) while simultaneously reporting strong feelings via self-report. Because the WEM postulates that emotions are, first of all, involuntary behaviors conveying a feeling, BPD patients should also demonstrate relatively strong muscle contractions in response to relatively low-level affective triggers. All of that is usually known as an “emotional storm” without disentangling the separate aspects, which the WEM defines. Interestingly, the same pattern of measures would be expected to show up in the case of slightly positive affective triggers.

Patients with MDD, on the other hand, should show a different scenario. By applying the WEM to MDD patients, one would expect positive affective triggers to elicit negative affection, which is due to the known negativity bias that has been mentioned above. At the same time, their conscious feelings might not align with their underlying affective processing responses, a condition that can lead MDD patients to self-reported positivity, while their measured affective processing demonstrates significant negativity. This approach demonstrates that integration in the brain does not mean that separate components are not linked to function as a unified whole, but rather highlights how these distinct layers of the WEM triad can be individually dysregulated in psychiatric conditions.

## 3. Comparative Analysis of the Walla Emotion Model and Traditional Theories

The WEM represents a paradigm shift from traditional theories that often conflate the various stages of affective phenomena. It is herewith proposed as an operational simplification as well as a broader theoretical redefinition. To help understand its clinical and research potential, it should be contrasted with the major frameworks that have historically dominated the field. The following list of mentioned theories is by no means exhaustive, but should be sufficient to demonstrate unwanted ambiguity related to the terminology of affective phenomena. Paul Ekman’s Basic Emotion Theory [35,36] is perhaps the most well-known, positing six universal, discrete categories: happiness, sadness, fear, anger, disgust, and surprise. Ekman treats these categories as “emotions” that have universal facial signatures. However, a direct relationship between a felt inner state and a respective facial expression is largely in question [37]. The WEM argues that “happiness” or “fear” should better be understood as feelings (conscious experiences), while only the facial expressions (among other behaviors) should be labeled “emotions” (behavioral outputs). Ekman’s model assumes a one-to-one correspondence between a feeling and an expression (the combination of which are called an emotion regarding this theory). The WEM acknowledges that these can be decoupled—a person can have the subcortical processing of fear without the conscious feeling or the facial expression. This is crucial for diagnosing disorders where expressions are masked or distorted, such as psychopathy or severe autism [12]. Ekman’s model is primarily behavioral/descriptive, whereas the WEM is explicitly neurobiological, focusing on a subcortical-to-cortical hierarchy following an evolution-based notion.

The also well-known Russell’s Circumplex Model is dimensional and posits that emotions are not discrete categories, but are points on a two-dimensional map of valence and arousal [38]. The WEM actually incorporates the dimensions of valence and arousal into its definition of affective processing. However, it critiques the use of these dimensions to describe “emotions”, which it understands as behaviors resulting from affective processing [1]. The Russell model often relies on self-report scales (e.g., the Self-Assessment Manikin). The WEM points out that these scales measure cognitively processed feelings and affective processing (i.e., 2nd-hand affection), not the raw affective state, which can only be accessed via objective metrics like Startle Reflex Modulation (SRM). While the Circumplex Model is excellent for mapping general mood states, the WEM is considered more useful for clinical interventions for various reasons.

While the Circumplex Model is well suited to describe how people feel (i.e., it is useful for self-reporting), the WEM is meant to add why people feel the way they feel. This distinction is considered critical in clinical psychopathology, because it means moving beyond symptom management toward addressing biological root causes. As mentioned above, many psychopathologies (like BPD and MDD) involve affective processing that never reaches consciousness or is mislabeled by the patient. The WEM allows clinicians to recognize that a patient’s “Emotion” (the biological output) might be highly active even if their “Feeling” (conscious awareness) is numb or disconnected, and how this relates to underlying affective processing. A major hurdle in clinical psychopathology is the gap between what a patient says and what their body does. The WEM acknowledges that sensory-driven reactions (affective processing) can lead to cognitively distorted interpretations (how the brain interprets bodily responses of raw affection into a feeling).

The James–Lange theory states that we feel an emotion because we perceive our body’s reaction (e.g., “I am trembling, therefore I am afraid”) [39]. Antonio Damasio’s Somatic Marker Hypothesis (SMH) expanded this, suggesting that bodily signals (“somatic markers”) bias our decision-making by acting as non-conscious “gut feelings” [40]. The WEM shares the somatic focus of James–Lange, but suggests an alternative nomenclature. In James–Lange, “emotion” is the perception of the body, whereas in WEM, that perception is a “feeling,” while the outward expression is the “emotion”. Damasio focused on how these signals help us make “rational” choices in complex tasks like the Iowa Gambling Task [41]. The WEM focuses more broadly on how affective processing guides all human behavior, including the impulsive, non-rational actions seen in clinical disorders like BPD. It explicitly defines the “supra-threshold” trigger for chemical release, which provides a more specific biological target than the broader “body loop” suggested by Damasio.

Lastly, some models include the cognitive domain. For example, the Schachter–Singer theory suggests that an emotion requires both physiological arousal and a cognitive label based on the environment [42]. The WEM emphasizes the primacy of affection and fundamentally challenges the necessity of cognition for affective states. It posits that affective processing occurs subcortically and is evolutionarily primary, which means that it does not need a label to drive behavior (in lower mammals as well as in very young humans behavior is mostly driven by affection). The WEM even highlights the potential cognitive pollution problem [11]. In clinical conditions like social anxiety, a patient might cognitively label their arousal as “fear” when it is actually “excitement,” or vice versa, based on social pressure or, simply, because language as a cortical function has only limited access to deeply subcortical information.

## 4. The Move to Objective Measures

One of the WEM’s most significant contributions to clinical methodology is its critique of self-report data. Since language is a cortical function and affective processing is subcortical, there is no direct neural pathway for an individual to accurately report their raw affective state. In other words, when affective processing evolved, language did not yet exist, which means that affective content was never meant to be verbalized. This access problem means that any diagnostic protocol based solely on interviews or surveys is possibly limited. In this essay, SRM has been mentioned several times because it indeed provides very good access to raw affective processing, independent from cognition and conscious interpretation. For this reason, the following section will shortly introduce this great tool that should be utilized more often. In combination with the WEM, it could indeed mean a methodological revolution.

### 4.1. Startle Reflex Modulation (SRM)

Startle Reflex Modulation (SRM) has emerged as a gold-standard tool within the WEM framework. By measuring the magnitude of the eye-blink reflex (an ancient survival mechanism) in response to a sudden noise while viewing stimuli that trigger affective processing, researchers can quantify raw affective valence without any conscious interference [43]. In clinical trials, SRM has revealed profound insights, for instance those related to childhood maltreatment [44]. Individuals exposed to childhood adversity show distinct defensive response profiles in SRM, even when their conscious self-reports are similar to healthy controls [44]. Further, SRM has been successfully used to measure non-conscious affection-related responses in patients with impulsive aggressive behaviors and schizophrenia, where self-monitoring is impaired [45]. In order to introduce SRM as a suggested method with great potential, its background shall be explained finally, leading to a short lab perspective in terms of what machinery is involved and how to use it as a tool to get access to raw affection.

#### 4.1.1. Brief SRM Background

**Figure 2:**
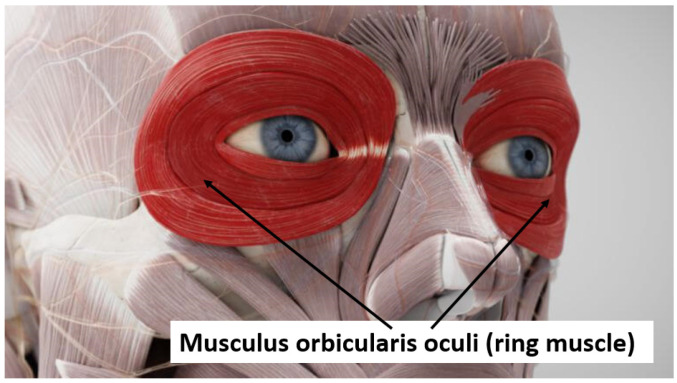
Musculus orbicularis oculi. Ring muscle around each eye that causes an eye blink when it contracts (adapted from https://flexikon.doccheck.com/de/Musculus_orbicularis_oculi, accessed on 30 April 2026).

In 1951, Brown et al. [46] measured startle responses to intense auditory stimuli with 30 rats, 15 of which underwent controlled fear conditioning during four consecutive days. The results showed a highly significant increase in the startle responses of fear-conditioned rats compared to control rats. The researchers used the heights of their jumps to the loud acoustic startle probes as measures of startle responses. The rats jumped higher to the constant startle probe when they were fear conditioned. The conclusion was that negative affection enhances the startle reflex. In principle, a startle response is a biological protection mechanism that consists of a complex pattern of muscle contractions, starting from the head and passing down more or less to the whole body [47,48]. While photographical techniques were first used to quantify the temporal dynamics of facial and skeletal muscle contractions [49], Jones & Kennedy (1951) [50] introduced electromyography (EMG) for the recording of startle patterns. In humans, it is the *musculus orbicularis oculi*, a ring muscle around each eye (Figure 2) which has the shortest latency and is most resistant to habituation [51]. This is why its contraction magnitude became the most common startle response measure in human experiments, with a short burst of acoustic white noise delivered at a sound pressure level of around 105 dB becoming the most common startle probe [52,53].

At this point, it does not seem very meaningful to measure the magnitude of a protective reflex response involving eye blinks to protect the eyes from potential harm. However, there is a crucial feature of the startle response that indeed makes it a highly reliable method to quantify raw affective responses. What had been mentioned before about rats jumping higher when they were fear-conditioned [46] translates very nicely to human beings. Even though it is an automatic brainstem-mediated reflex, it is subject to modulation, which means that its magnitude is dynamically changing depending on the state of affection. In the animal literature, one can find that those changes are caused by facilitatory neural projections from the amygdalae to the brainstem nucleus known as nucleus reticularis pontis caudalis (it is part of the acoustic reflex neural circuit) [54] and also by inhibitory projections from the nucleus accumbens. When the level of amygdalae activation increases because it detects negative content, this potentiates the startle response. Amygdalae lesions were indeed shown to impair the potentiation of the startle response [55], and fear extinction through amygdalae influence could be measured via Startle Reflex Modulation [56]. On the other hand, when the level of nucleus accumbens activity increases because it detects positivity, this reduces the startle response [57,58]. Lesions of the nucleus accumbens block this inhibition [59]. The above-mentioned nucleus reticularis pontis caudalis [54] is a target of inputs from various brain nuclei, including the nucleus accumbens, and can be seen as a sensorimotor interface, where excitatory and inhibitory afferent connections can influence motor output [60]. What had first been found in animals importantly translates to humans. Simons & Zelson [57] successfully showed that presentations of pleasant pictures to human subjects while startling them diminishes respective startle responses, while presentations with negative content enhances them [61]. Already, at this time, it has been suggested that the probe startle paradigm may be readily put to use in a wide variety of psychophysiological laboratories.

By today, it has gained widespread acceptance and has become a highly appreciated research standard. As already mentioned above, because of reliable elicitation, the most commonly used stimulus type to elicit startle responses in humans is an acoustic white noise probe of high intensity (around 105 dB) and short duration (usually only 50 m) [62].

Possible applications are more or less unlimited. Thereby, the magnitudes of eye blink responses (EMG amplitude signal) elicited under controlled startle probe delivery are the measures that represent the dependent variable. Once this measure can be taken with an adequate EMG and startle probe delivery setup, one can freely choose a set of independent variables and design an experiment that enables the comparison of different experimental conditions with respect to their affective impact. In other words, it is then possible to quantify affective responses in the human brain to almost anything that stimulates at least one of the sensory systems. In the past, controlled acoustic [63], visual [64], olfactory [65], and even taste stimuli [53] have been used within SRM experiments to measure affective responses to those stimuli. The most crucial point is that no verbal responses need to be recorded. However, they can be recorded and compared with the raw affective responses (SRM data) to eventually find discrepancies. The independency of SRM data from explicit, verbal responses is of particular interest, because self-reported responses to affective content can be “cognitively polluted”, as has been mentioned above [11]. In other words, comparisons between self-reported affective responses (that could be explicitly stated likes or dislikes) and SRM data can reveal differences between survey results and actual affective impact.

#### 4.1.2. Methodological Setup and Experimental Design

SRM is, in fact, a quite straightforward method that can be used for hypothesis-driven, as well as rather exploratory experiments. As pointed out earlier, affective processing, which takes place deeply subcortically, was never meant to be verbalized and is separate from language (which is as a cortical function). Language often fails to correctly interpret raw affection, leading to possible discrepancies between SRM data and self-reports. Actually, exactly those are the most interesting, because they highlight that the brain indeed knows more than it admits to the consciousness.

**Figure 3:**
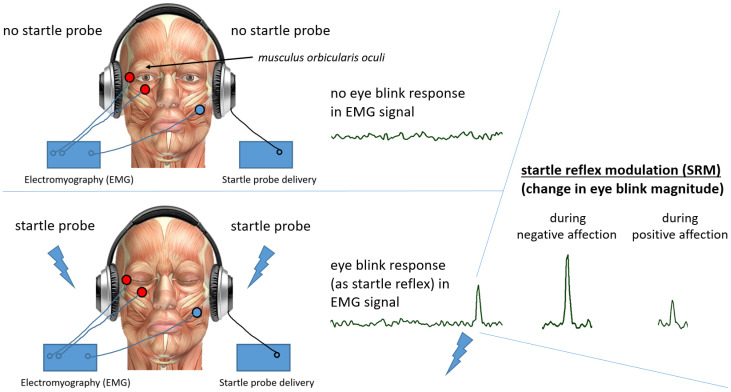
Methodological setup including face muscle anatomy, showing the relevant ring muscle around each eye (musculus orbicularis oculi). A standard SRM setup including EMG (3 electrodes) to record muscle potential changes when the ring muscle around an eye contracts to quantify eye blink responses to an acoustic startle probe delivered via a set of headphones. The startle probe is a 50 ms long burst of white noise delivered at around 105 dB.

The components for an SRM experimental setup are fairly easy to get and put together. Obviously, a physiological recording device is needed to collect EMG data from one of the ring muscles around the eyes (see Figure 3). Further, a PC or laptop computer is needed for physiological data collection. Depending on the physiological recording device one has available and on the design of the experiment, a further PC or laptop might be needed for the presenting of controlled foreground stimulation (i.e., the independent variables). For instance, a set of independent variables, such as pictures or words of different valence categories, is presented in a random order, while the dependent measure is recorded in response to the presentations of from time to time occurring short startle probes.

Imagine looking at a picture that shows a mutilated human body, which, obviously, is processed as a very negative stimulus. Further, imagine seeing it for 6 s, whereby somewhere within the sixth second you are startled by a loud shotgun-like sound. The loud sound elicits an eye blink response and its magnitude is recorded and quantified via EMG. Now, imagine seeing an image of a cute baby, while again being startled somewhere within the sixth second, and getting your respective eye blink response recorded. If your brain processed the cute baby image more positively than the mutilated body image, which would be expected, then your eye blink response to the loud short startle probe would be larger in the case of the mutilated body compared to the baby image. In summary, one gets more easily and strongly startled while the brain processes negativity than when it processes positivity (like how you are easier to startle while watching a horror movie compared to a comedy).

From the data perspective, the raw muscle potential signal is a high frequency signal, which is then calculated into an amplitude signal (e.g., via the root mean square method; see Figure 3). In short, the stronger the contraction of the ring muscle around the eye, the larger the amplitude (i.e., eye blink magnitude).

When designing a SRM experiment, one needs a few things to pay attention to. Let us say that the research question is focused on a comparison of raw affective responses elicited by two different products, like two smartphones from different brands. In such a case, one can actually let study participants play around with each phone separately for 5 min, while, during those 5 min, four startle probes are delivered through headphones. The time interval between the startle probes should not be constant, but varying. The minimal time between startle probe deliveries should be around 40 to 50 s, which is due to potential habituation. One can then calculate an average across the respective four startle responses for each phone and, finally, compare these numbers for a larger sample of participants. Alternatively, one could rather focus on visual stimulus variations belonging to certain categories, such as negative images versus positive images. In this case, one could select a set of 30 negative and 30 positive images and present each image for 6 s in random order. Each image should be preceded by a black screen (1 s), and the black screen by a fixation cross on a black background (1 s). One could then associate 5 of the 30 images per category with a startle probe. In fact, anything that stimulates at least one of the senses can be a controlled foreground stimulus. Finally, it has to be mentioned that SRM is a completely non-invasive method that allows one to quantify raw affective processing around cognition without the need for any self-report. It is considered superior to other brain imaging tools and possibly best suited in the context of clinical psychopathology.

## 5. Limitations

While the WEM emphasizes the important distinction of raw affection and subjective experiences and emotion behaviors, its primary limitation in a clinical setting is the difficult access to the level of non-conscious affection. Accessing this level of information processing requires the use of technology and its respective training. Clinicians generally lack the neuroimaging or physiological monitoring tools required to quantify these components in a standard therapy session. Additionally, by de-emphasizing feelings as the only conscious part of the triad, there is a risk of under-prioritizing the patient’s subjective narrative, which remains the primary information source of most therapeutic alliances. The WEM has a quite radical nature and, if implemented more dominantly, would require widespread adaptations. For instance, most clinical diagnostic manuals (like the DSM-5) and patients use the terms of the WEM interchangeably. A clinician using the WEM’s strict terminology may find it difficult to communicate with insurance providers, other medical professionals, or the patients themselves without constant translation and explanation. Even though the WEM’s terminology is proposed in order to simplify communication, it could, at least in the beginning, lead to more confusion than clarification.

## 6. Conclusions

The WEM advocates for a future where clinical psychology and psychiatry are grounded in the objective measurement of brain function in combination with the subjective report of symptoms. By narrowing the definition of “emotion” to observable behavior and “feelings” to conscious perception, both resulting from non-conscious affective processing, the model removes the ambiguity that has plagued the field for over a century. This reductionist yet potentially helpful approach might become essential for identifying biomarkers of mental health, and their respective disordered conditions. If we can identify the specific “affective processing” profiles associated with conditions like BPD, childhood maltreatment, or chronic depression, we can move toward preventative interventions that target the subcortical roots of these disorders. Ultimately, this model serves as a methodological bridge, amending the cognitively polluted data of self-report in psychology and psychiatry with objective, biological measurement of the human psyche. In doing so, it provides clinicians with the tools to better understand, diagnose, and treat the complex spectrum of affective disorders that define the human condition.

## Figures and Tables

**Table 1:** Summary of the three WEM components.

Component	Biological Locus	Function	Measurement	Access to Consciousness
Affective Processing	Subcortical/Limbic System	Raw evaluative decision-making (valence/Arousal)	Neurophysiological recording/Startle Reflex Modulation (SRM)	Non-conscious
Feelings	Cortical/Periphery	Subjective perception of supra-threshold bodily changes	Self-report/questionnaire	Conscious/subjective experience
Emotions	Motor System/Muscles	Communication of internal states to conspecifics	Electromyography (EMG)/film and photograph	Observable/behavioral
